# 

**Published:** 1909-09-25

**Authors:** 


### CHARITABLE BEQUEST.

The Rev. Edward Llewellyn Adams, of Scarborough, Congregational minister, for many years pastor of the Eastborough Congregational Church, who died in June, aged 80, and left estate of upwards of £34,400, bequeathed £500 to the Smedley Memorial Hydropathic Hospital for the endowment of a ward. Various other charitable legacies under the will were all reduced by a codicil in consequence of the added charges proposed in the present Budget. The testator stated: "It is with great sorrow that I make them [the reductions], but I am compelled to do so on account of the proposals in regard to the estate and legacy duties made in the Budget of April 29, 1909. I consider them to be most unwise and unjust. Unless I do so I find I shall not leave sufficient money to pay the various legacies left under my will." The revised bequests include the following:—£4,000 (reduced from £4,500) to the New Manchester Royal Infirmary for an "Elizabeth Adams" Ward; £4,000 (reduced from £4,500) to the chancellor and treasurer of the Victoria University of Manchester for founding and endowing a commercial scholarship or scholarships to be called the "Charles John Adams" Scholarship or scholarships; £4,000 (reduced from £4,500) to the Scarborough Hospital for the permanent endowment of a children's ward, but charged with a life annuity of £10 to be paid to Frances Ward.

### THE GRADUATES' MEDICAL SCHOOLS.

DIARY FOR SEPTEMBER 27 to OCTOBER 2.

### THE WEST-END HOSPITAL FOR NERVOUS DISEASES, Welbeck Street, W.

**Clinical Demonstrations.**

**At 1.30 p.m.**
**September 27,** Dr. Harry Campbell.

**At 5.30 p.m.**
**September 28,** Dr. James Mackenzie.

**At 1.30 p.m.**

**September 29,** Dr. Frederick S. Palmer.

**September 30,** Dr. T. D. Savill.

**October 1,** Dr. Purves Stewart.

**At 5.30 p.m.**

**October 1,** Dr. Eric D. Macnamara.

### MEDICAL GRADUATES' COLLEGE AND POLYCLINIC, 22 Chenies Street, W.C.

**Clinique.**

**At 4 p.m.**
**September 27,** Dr. J. L. Bunch, **Skin.**
**September 28,** Dr. Leonard Guthrie, **Medicine.**
**September 29,** Mr. Cecil Leaf, **Surgery.**
**September 30,** Sir Jonathan Hutchinson, **Surgery.**
**October 1,** Mr. J. Gay French, **Ear, Nose, and Throat.**

### POST-GRADUATE COLLEGE, WEST LONDON HOSPITAL, HAMMERSMITH, W.

The special arrangements for the ensuing winter session are as follows :—There will be a lecture or demonstration every day (Saturdays excepted) at 5 P.M. Amongst these will be : (1) Six lectures on Medicine, by Dr. A. P. Beddard, on Wednesdays, Oct. 6, 20, 27, Nov. 10, 17, and Dec. 1. (2) Six lectures on Practical Surgery, by Mr. Aslett Baldwin, on Mondays Oct. 25, Nov. 15, 22, 29, Dec. 6 and 13. (3) Three lectures on *x*-Rays, illustrated by lantern slides, by Dr. Reginald Morton, on Wednesdays, Oct. 13, Nov. 16, and Tuesday, Dec. 14. (4) Three lectures on Mental Diseases, by Dr. Robert Jones, on Tuesdays, Oct. 12 and Dec. 7, at 5 P.M. in the Lecture Room; and Tuesday, Nov. 23 (at the London County Asylum, Claybury, Woodford Bridge, Essex, at 3 P.M.). (5) Three lectures on Tropical Medicine, by Dr. G. C. Low, on Wednesdays, Nov. 3, 24, and Dec. 8.

Lectures on Practical Medicine will be held in the Lecture Room at 12.15 P.M. on Tuesdays by Dr. Harold Pritchard, and on Wednesdays and Saturdays at 12.15 P.M. by Dr. Grainger Stewart. Demonstrations on Cases in the Medical Wards will be held on Fridays at 10 A.M. by the Medical Registrar (except Oct. 8 and 15). Demonstrations on Cases in the Surgical Wards will be held on Mondays and Thursdays at 10 A.M. by the Surgical Registrar. Pathological demonstrations will be held in the laboratory on Thursdays at 12 noon by Dr. Bernstein, the pathologist.

In addition to the above, demonstrations on Cases in the Wards will be given as follows by the physicians and surgeons :—Dr. Seymour Taylor—Fridays : Nov. 12 and Dec. 3; and Tuesdays : Nov. 16 and Dec. 7, at 3 P.M. Dr. Beddard—Saturdays : Oct. 9, 30, Nov. 20, and Dec. 11, at 2 P.M. ; and Wednesdays : Oct. 13, Nov. 3, 24, and Dec. 15, at 4.15 P.M. Dr. Saunders—Thursdays : Oct. 14, Nov. 4, 25, and Dec. 16; and Mondays : Oct. 18, Nov. 8, and 29, at 3 P.M. Mr. Keetley—Thursdays : Oct. 21, Nov. 11, and Dec. 2, at 1.45 P.M. Mr. Edwards—Thursdays : Oct. 7, 28, Nov. 18, and Dec. 9, at 4 P.M. Mr. Bidwell—Mondays : Oct. 11, Nov. 1, 22, and Dec. 13, at 3 P.M.

Demonstrations are given daily in the medical and surgical out-patient rooms and in the special departments at 2 P.M. The physicians and surgeons attend daily at 2.30 P.M. Special classes will be formed for instruction in Diseases of the Throat, Nose and Ear, the Skin, and the Eye ; *x*-Rays ; Gynæcology ; the Administration of Anæsthetics ; the Clinical Examination of Blood and Urine ; Bacteriology ; Medical Electricity ; Microscopy ; Pathology ; Surface Anatomy ; Intestinal Surgery ; Operative Surgery on the Cadaver ; Operative Ophthalmic Surgery ; Tropical Medicine ; Cystoscopy ; Venereal Diseases ; and Children's Diseases. A special class in Bacteriology (consisting of twelve demonstrations) is held every month by Dr. Bernstein, and meets on Tuesdays and Fridays at 11.30 A.M. and on Thursdays at 10.30 A.M. The fee for the attendance on the hospital practice for three months (including all ordinary lectures and demonstrations) is £6 6s.

